# Diagnosis of Idiopathic Fevers

**Published:** 1868

**Authors:** F. M. Brantly

**Affiliations:** Rocky Mount, Ga.


### ARTICLE II.

*Diagnosis of Idiopathic Fevers.* By F. M. Brantly, M. D., of Rocky Mount, Ga.

The great desideratum in the successful treatment of disease, is an early and correct diagnosis. Although most of the maladies that are incident to the human family may be rationally and scientifically treated on general principles, yet "the more perfect and sure way" is to understand the pathological and anatomical lesions, or, in other words, to be able to locate and identify the true source of each particular affection. Many diseases will not permit delay in their diagnosis, without great risk to the patient. Hence the difference in the success of Doctors; some are ever learning and never come to a knowledge of the truth, while others appear to arrive at correct conclusions almost without an effort.

With regard to the etiology of fevers, I am inclined to the opinion that there are but two principal causes, and that these two causes produce two distinct kinds of fever. The combination of these causes gives rise to mixed or mongrel fevers, and it is this class that has wrought such havoc in our nomenclature. One might suppose, from reading the various authors extant, that there was really a vast number of distinct idiopathic fevers, but to sum up the whole matter according to etiological and pathological principles, the conclusion is forced upon the mind that a vast amount written upon this subject is mere hypothesis, and likely to be erroneous.

The causes above alluded to are, vegetable malaria and animal miasm, the former producing intermittent, remittent, billious, congestive, and all that kind of fevers that have marked exacerbations, and the latter producing that class of continued fevers commonly known as typhus, typhoid, synochus, synocha, enteric, &c. From this, it would seem that there are really but two kinds of distinct idiopathic fevers to be met with in this country. This we believe to be the truth, and for the sake of simplicity and clearness, we prefer to apply the terms remittent and continued, as expressing our idea of these two classes.

From our classification, it would seem an easy task to refer any case to its proper place, but what would appear easy in theory is often difficult in practice; for it often happens, that one of these orders so nearly assimilates the other, owing to fortuitous circumstances, that we scarcely know whether we should call it remittent or continued. Doubt less many lives have been sacrificed by want of this discrimination, because the treatment that would be proper in the one case would often be extremely pernicious, and even fatal, in the other. We should never lose sight of the idea that the two classes under consideration possess a separate identity, lest we find Scylla upon the one hand and Charybdis upon the other.

We will now proceed to mark some of the differences between remittent and continued fevers; and here, one of the first essentials is a good time-keeper, because it is mainly to the action of the heart and arteries that we must look, as the most certain index for a proper discrimination. In remittent fevers, the pulse will often take the widest range, both as to frequency and volume, sometimes varying from twenty-five to fifty beats per minute in twenty-four hours, and sometimes being full and bounding, and then soft and yielding. Alimentary evacuations, either with or without medicines, have a tendency to lessen, for a time, the frequency of the heart's action. In like manner will all the other signs exhibit marked exacerbations, being better one day and much worse the next. This class, for the most part, yields readily to medical treatment.

Continued fever, unlike the foregoing, is usually insidious in its approach, producing, at first, oppression and dulness in the base of the brain, with lethargy and general langour of the whole system. The pulse remains frequent, varying not more than from five to ten beats in the twenty-four hours. Although there may be no external indications of fever in the morning, yet upon examination, the frequency of the pulse is generally found to be about the same as when there is every external sign of fever. In the absence of the exacerbations, the pulse may lose some in volume, but rarely much in frequency. There is another constant and reliable symptom, of great importance in the diagnosis of this disease. After each alimentary evacuation, and especially if it be brought about by purgatives, the pulse will suddenly increase in frequency, and so remain for a long time, and only gradually subsiding by quiet, and a suspension of the alvine evacuations; and the longer this suspension lasts, the nearer will the pulse approach a normal standard, provided the torpidity does not continue an unreasonable time.

By a careful observation of the above named symptoms, together with those usually enumerated in works on diagnosis, much precious time may be saved, thus greatly enhancing the chances for life in the treatment of continued fever, as much depends upon the *early* application of proper remedies and regimen. A large majority of the fatal cases occurring in this disease are due mainly to improper treatment *before* the nature of the disease is clearly understood, and to a too free use of medicines *after* its true character is apprehended. This disease cannot be cut short by any remedial agents with which we are acquainted. The animal miasma, the exciting poison, must be expelled, by physiological forces, from the body. Those persons in whom the functions of absorbtion and elimination are most active, will recover soonest, and *vice versa*. In my humble judgment, the great fatality heretofore attending this disease, is dependent, not upon the disease itself, or anything essentially belonging to it, but upon adventitious circumstances, favored by the ennervating influences of the disease.

Continued fever is, to a certain extent, infectious, often extending through whole families, and to others who remain long near the sick.

